# Use of the GlideScope video laryngoscope for intubation during ex utero intrapartum treatment in a fetus with a giant cyst of the 4th branchial cleft: A case report

**DOI:** 10.1097/MD.0000000000004931

**Published:** 2016-09-30

**Authors:** Sung Hye Byun, So Young Lee, Seong Yeon Hong, Taeha Ryu, Baek Jin Kim, Jin Yong Jung

**Affiliations:** a Department of Anesthesiology and Pain Medicine; b Department of Obstetrics and Gynecology, School of Medicine, Catholic University of Daegu, Daegu, Republic of Korea.

**Keywords:** ex utero intrapartum treatment, fetal neck mass, GlideScope, video laryngoscope

## Abstract

**Introduction:**

In fetuses who are predicted to be at risk of catastrophic airway obstruction at delivery, the ex utero intrapartum treatment (EXIT) procedure is useful for securing the fetal airway while maintaining fetal oxygenation via placental circulation. Factors, including poor posture of the fetus and physician, narrow visual field, and issues of contamination in the aseptic surgical field, make fetal intubation during the EXIT procedure difficult. Herein, we report our experience of the usefulness of the GlideScope video laryngoscope (GVL) for intubation during the EXIT procedure.

**Symptoms and clinical findings:**

A 28-year-old woman presented with a fetus having a cystic neck mass diagnosed on prenatal ultrasound at 25 weeks of gestation. We planned the EXIT procedure in conjunction with cesarean delivery at 38 weeks of gestation, as the mass enlarged to 4.9 cm × 3.2 cm, protruded externally at the neck, and subsequently resulted in polyhydramnios.

**Therapeutic intervention and outcomes:**

After induction of anesthesia using intravenous thiopental (300 mg), adequate uterine relaxation was achieved with sevoflurane (2.0–3.0 vol%) combined with continuous intravenous infusion of nitroglycerin (0.5–1.0 μg/kg/min) for maintaining uteroplacental circulation. After hysterotomy, the head and right upper limb of the fetus were partially delivered, and fetal heart tones were monitored with a sterile Doppler probe. After oropharyngeal suctioning to improve the visual field, the fetus was intubated successfully using a sterile GVL by an anesthesiologist, and the passage of the endotracheal tube beyond the vocal cords was confirmed on the screen of the GVL system. Immediately after the fetal airway was definitely secured, the fetus was fully delivered with umbilical cord clamping. After delivery, nitroglycerine administration was ceased and sevoflurane administration was reduced to 0.5 minimum alveolar concentration. Additionally, oxytocin (10 units) and carbetocin (100 μg) were administered for recovery of uterine contraction. Cesarean delivery was successfully performed without any problems, and the neonate successfully underwent surgery for removal of the neck mass under general anesthesia on the 7th day after delivery. The neonate is developing normally.

**Conclusion:**

The GVL approach may be a useful noninvasive approach for establishing a clear fetal airway during the EXIT procedure.

## 1 Introduction

The ex utero intrapartum treatment (EXIT) procedure is a useful technique at delivery in cases in which neonatal airway difficulty and subsequent respiratory failure are anticipated because of a fetal congenital disease that has been diagnosed prenatally.^[1–3]^ With maintenance of fetal oxygenation via uteroplacental circulation, sufficient time is provided to achieve airway access in a fetus through direct laryngoscopy, fiberoptic bronchoscopy, or tracheostomy.^[4–7]^ However, it is very challenging to establish airway access during the EXIT procedure, even for anesthesiologists who are very skilled at airway management, because such a fetus has severe airway difficulty that necessitates the EXIT procedure and there is insufficient room for airway management within the surgical field. To facilitate management in difficult airway cases, the GlideScope video laryngoscope (GVL; Verathon Medical, Bothell, WA) was recently introduced. The GVL consists of a 60° angulated blade and a digital camera located at the distal end of the blade to visualize airway structures without the need for direct line-of-sight in order to achieve tracheal intubation.^[8]^ With regard to pediatric patients, the GVL has been reported to provide a better view of the glottis in neonates and infants,^[9]^ and the performance of tracheal intubation has been reported to be better with the GVL than with a direct laryngoscope in older children with a difficult airway.^[10,11]^ Herein, we report our experience of the usefulness of the GVL for intubation during the EXIT procedure in a fetus with a giant cyst of the 4th branchial cleft that was expected to cause neonatal airway-related issues, such as hypoxia, brain injury, and even death, associated with airway obstruction.

## 2 Case presentation

**Figure 1:**
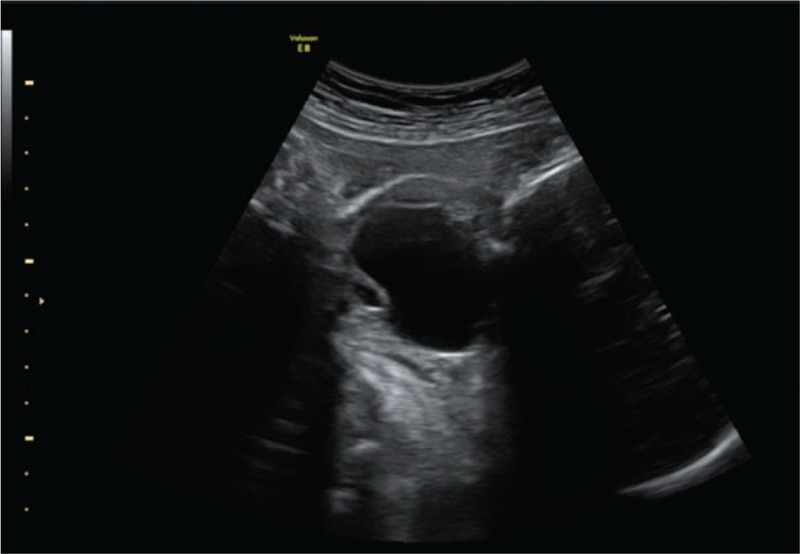
A cystic lesion (size, 4.9 cm × 3.2 cm) is seen in the anterolateral region on the left side of the neck on ultrasound performed at 36 weeks of gestation.

A 28-year-old healthy pregnant woman (height, 160 cm; weight, 62 kg; gravida, 2; abortion, 2) was referred to our institution for prenatal care at 25 weeks of gestation because a prenatal ultrasound showed that the fetus had a cystic neck mass. The mass was 2.2 cm in length, and it extended from the base of the tongue to just above the thoracic cavity in the anterolateral region on the left side of the neck. The patient had no relevant medical or family history, and there was no evidence of congenital anomalies in the prenatal genetic screening. On ultrasound performed at 36 weeks of gestation, the fetal neck mass was found to have enlarged to 4.9 cm × 3.2 cm in size, and it protruded externally at the neck and subsequently resulted in polyhydramnios (Fig. 1). Therefore, elective cesarean delivery and the EXIT procedure were scheduled at 38 weeks of gestation. A multidisciplinary team, including obstetricians, anesthesiologists, neonatologists, otolaryngologists, and assistant nurses, was organized for the EXIT procedure.

On the day of surgery, the mother was placed in the left uterine displacement position to avoid aortocaval compression on the operating table, and large-bore venous access was established in both arms beforehand. Standard monitoring procedures, including electrocardiography and pulse oximetry, were conducted, and radial arterial cannulation was performed for continuous blood pressure monitoring before induction of general anesthesia. Following adequate preoxygenation, rapid sequence induction was performed with administration of intravenous thiopental (300 mg) and succinylcholine (62 mg). After tracheal intubation was performed using a 6.5-mm cuffed endotracheal tube (ETT), a muscle relaxant (rocuronium, 60 mg) was administered intravenously. General anesthesia was maintained with sevoflurane (2.0–3.0 vol%) in an O_2_/N_2_O mixture (FiO_2_ 0.5, 4 L/min) and liberal intermittent intravenous administration of a bolus of fentanyl. For adequate relaxation of the uterus until the umbilical cord was clamped and the fetus was fully delivered, the end-tidal concentration of sevoflurane was maintained at approximately 1 to 2 minimum alveolar concentration (MAC). In addition, intravenous nitroglycerin was infused at a rate of 0.5 to 1.0 μg/kg/min to enhance uterine relaxation. Moreover, intravenous infusion of dopamine was used to maintain maternal blood pressure.

**Figure 2:**
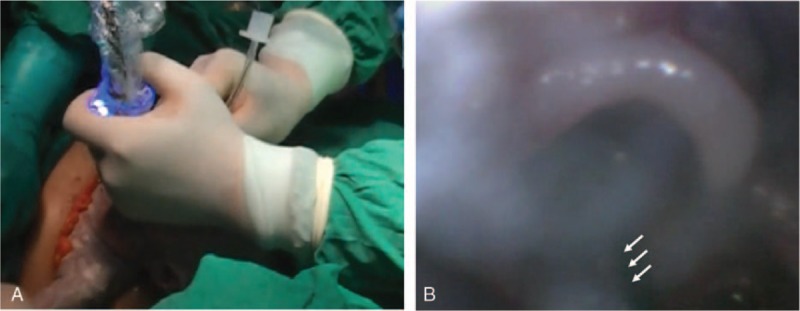
The EXIT procedure. (A) Using the GVL, the fetus is intubated successfully with a noncuffed ETT having an internal diameter of 3.0 mm and a stylet. (B) Passage of the ETT beyond the vocal cords is confirmed on the screen of the GVL system. The white arrow indicates the ETT. ETT = endotracheal tube, EXIT = ex utero intrapartum treatment, GVL = GlideScope video laryngoscope.

After surgical exposure of the uterus, the obstetricians confirmed adequate uterine relaxation, and hysterotomy was performed. After the head and right upper limb of the fetus were partially delivered while preserving the uterine volume with prevention of uterine contraction, a sterile oximetry probe was applied to the right hand of the fetus by an assistant obstetrician. However, the pulse wave signal was not being well detected with the oximetry probe, and therefore, fetal heart tones were monitored with a sterile Doppler probe until the fetal airway was secured. Before fetal intubation, the video baton of the GVL was draped with sterile vinyl cover and was equipped with a sterile single-use blade (size 1 for 1.5–3.6 kg newborns) beforehand for manipulation within the aseptic surgical field. The staff anesthesiologist (JYJ), who was wearing a surgical gown and gloves, attempted intubation of the fetus using the GVL (Fig. 2A), and the fetal airway structure was observed on the screen of the GVL system. After careful oropharyngeal suctioning to improve the visual field, the fetus was intubated successfully using a noncuffed ETT (internal diameter, 3.0 mm) with a stylet, and passage of the ETT beyond the vocal cords was confirmed on the screen of the GVL system (Fig. 2B). Immediately after the fetal airway was definitely secured and adequate positive-pressure ventilation was feasible, the fetus was fully delivered with umbilical cord clamping. The total time from hysterotomy to full delivery of the fetus was approximately 5 minutes. After delivery, the oxygen saturation value was 99% and the fetal heart rate was 140 beats/min (assessed with the pulse oximetry probe on the hand of the fetus). The neonate had a 1-minute Apgar score of 8 and a 5-minute score of 10. The neonate was transferred to the neonatal intensive care unit by neonatologists while maintaining self-respiration, without the need for artificial ventilation.

After the completion of delivery, the nitroglycerine and dopamine infusions were ceased and sevoflurane administration was reduced to 0.5 MAC. Then, an intravenous bolus of 10 units of oxytocin was injected, followed by slow infusion of 100 μg of carbetocin (long-acting oxytocin receptor agonist) that was added to 500 mL of colloid, in order to obtain adequate uterine contraction for prevention of severe postpartum hemorrhage. The total time of surgery was 157 minutes, and the total estimated blood loss was 700 mL. Additionally, the total administration of crystalloid was 600 mL and that of colloid was 500 mL during the surgery. The mother was extubated uneventfully after the completion of surgery and recovered well in the postanesthetic care unit. On the 7th day after delivery, the neck mass of the neonate was surgically removed by an otolaryngologist under general anesthesia, owing to abrupt enlargement of the mass, with abscess formation. The mass (size, 5.5 cm × 4.0 cm × 0.4 cm) had marked inflammation and was confirmed to be an infected cyst of the 4th brachial cleft based on the anatomical location and pathological findings. Approval was not needed from the institutional review board as the clinical data were deidentified, and written informed consent was obtained from the patient for the publication of this case report.

## 3 Discussion

The EXIT procedure is an uncommon technique, but it is indicated in fetuses with life-threatening airway obstruction owing to a congenital disease or malformation at the time of delivery.^[1–6,12]^ Recent technological advancements in prenatal diagnosis have allowed the early detection of fetal malformations before delivery, thereby facilitating planning of appropriate treatment for such cases and improving the perinatal outcome.^[7]^ In fetuses who are expected to have airway difficulty and subsequent airway crisis related to airway obstruction at delivery, the EXIT procedure has been reported to be appropriate for securing the fetal airway.^[2–7]^ Lazar et al^[7]^ reported that the EXIT procedure for a giant neck mass could reduce the mortality rate to 8%, when compared to the mortality rate of 10% to 57% reported in infants not undergoing the EXIT procedure.^[13–15]^ Additionally, Lazar et al^[7]^ reported that only 1 infant in their series had significant neurodevelopmental delay, while historically, up to 20% of neonates with giant neck masses have been reported to have neurodevelopmental delay due to suspected hypoxia at the time of traditional delivery.^[7]^ The EXIT procedure is performed during cesarean section, and the approach involves partial delivery of the head and upper limb of the fetus and maintenance of uteroplacental circulation until the airway of the fetus is established definitely. As fetal oxygenation is continuously maintained with uteroplacental circulation during the EXIT procedure, the physician has sufficient time to achieve airway access in the fetus. When it appears possible to secure the fetal airway with conventional direct laryngoscopy, this should be the first-choice technique for airway management.

However, it is very challenging to establish a clear airway during the EXIT procedure owing to various reasons, even for anesthesiologists who are very skilled at airway management. The fetus usually has severe airway difficulty that necessitates the EXIT procedure. Distorted anatomy of the fetal airway should be strongly suspected in fetuses with a large neck mass, as in our case. A large lesion in the neck can compress the surrounding cervical structures, thereby causing impaired fetal swallowing, polyhydramnios, or preterm labor, and more specifically fetal airway obstruction, neonatal hypoxia, or death.^[7]^ For establishing a clear fetal airway safely and effectively during the EXIT procedure, various modalities can be used by anesthesiologists or neonatologists, including direct laryngoscopy and fiberoptic bronchoscopy. If a clear fetal airway cannot be achieved with these modalities, surgical tracheostomy can be performed as the last option by otolaryngologists or pediatric surgeons.^[4–7]^ Bouchard et al^[4]^ presented their experience with 31 cases of the EXIT procedure. The authors reported that 77% (24/31) of the fetuses were intubated successfully after direct laryngoscopy or rigid bronchoscopy, whereas 6 fetuses could not be intubated, even using rigid bronchoscopy, and eventually required surgical tracheostomy. Lazar et al^[7]^ reported that only 1 fetus could be intubated with direct laryngoscopy alone, 10 fetuses required rigid bronchoscopy, and 1 fetus required tracheostomy among 12 fetuses that underwent the EXIT procedure. Considering these results, conventional direct laryngoscopy alone has limitations for routine use and even rigid bronchoscopy has limitations as a final nonsurgical option. Nevertheless, noninvasive approaches of intubation should be attempted in cases of difficult intubation before moving to an invasive approach, such as tracheostomy. In addition to the inherent difficulty with regard to the fetal airway, there is the problem of insufficient space for airway management within the surgical field during the EXIT procedure. As the fetus is partially externalized from the uterus, it cannot be placed in the sniffing position, which is considered the optimal head position for direct laryngoscopy. Therefore, the body posture of the physician when performing fetal intubation during the EXIT procedure is unusual and inconvenient. Additionally, because of the narrow visual field within the small oropharyngeal cavity of pediatric patients, physicians usually tend to bend their trunk or neck more than usual and approach close to the pediatric patient when intubating. Fetal intubation is performed in a sterile surgical field, and the physician attempting intubation of the fetus should not contaminate the surgical field. Consequently, these above-mentioned factors make tracheal intubation of the fetus during the EXIT procedure more difficult. In fact, in our institution, difficulty with a direct laryngoscope has been experienced in a previous case involving the EXIT procedure, and tracheal intubation was successful after multiple attempts by neonatologists. Accordingly, the GVL was used as the primary option for securing the fetal airway during the EXIT procedure in the present case.

The GVL is designed to allow viewing of the glottis with a digital camera and specially curved blade, without alignment of the oral, pharyngeal, and laryngeal axes. The 60° angulation of the blade and the presence of the camera at the distal end of the blade provide a more anterior laryngeal view, thereby improving the glottis view when compared with the view obtained with a direct laryngoscope.^[8]^ For the management of difficult airway in adults, video laryngoscopes (VLs) are superior to direct laryngoscopes with regard to the laryngeal view and intubation success rate.^[16]^ Recent publications have demonstrated that the glottis view is better and the time taken to obtain the best glottis view is shorter with GVLs than with direct laryngoscopes in neonates and infants with an anatomically normal airway,^[9]^ as well as older children with a difficulty airway.^[10,11]^ However, the intubation time and success rate were similar between the GVLs and the direct laryngoscopes.^[9–11]^ In our case, the staff anesthesiologist could achieve fetal intubation in the first attempt using the GVL, within about 5 minutes after hysterotomy. Successful tracheal intubation in this fetus, who had a potentially difficult airway and was placed in an unfavorable position for tracheal intubation, was possible because the GVL allowed the physicians to observe the fetal airway structure clearly and confirm the passage of the ETT beyond the vocal cords definitely, without the need for the sniffing position.

With regard to the body posture of the physician, the GVL has some advantages over the direct laryngoscope. Grundgeiger et al^[17]^ demonstrated that the use of the GVL resulted in less trunk and neck flexion and less extended upper arms, namely small deflections of the posture angles, for experts. More ergonomic body posture may be facilitated because the GVL rarely requires the physician to look directly into the patient's mouth for viewing the larynx.^[17]^ Moreover, when using the GVL, the posture of the physician is more consistently erect and the physician is not close to the patient, thereby reducing the risk of contamination of the sterile surgical field.

There are some considerations regarding the use of the GVL during the EXIT procedure. First, the physician who will use the VL for patients with a difficult airway in awkward situations should become accustomed to manipulation of the VL under ordinary circumstances of tracheal intubation. A VL with an angulated blade, which is similar to the GVL, may cause difficulties when an inexperienced physician attempts to advance the blade into the oral cavity or insert the ETT into the trachea, and therefore, a lot of practice and experience with a VL would be needed. A recent meta-analysis noted that a prolonged time to intubation with a VL is probably associated with the relatively low experience of the physician with a pediatric VL, and this prolonged time consequently increases the rate of a failed first intubation attempt in pediatric patients.^[18]^ Therefore, this approach should be recommended with caution in children, especially those who may not tolerate long-term apnea.^[18]^ Fortunately, as adequate fetal gas exchange is maintained with uteroplacental circulation, the duration of tracheal intubation is believe to not be an issue during the EXIT procedure in contrast with standard cesarean delivery, and the length of placental support has been reported to vary depending on the interventions performed in the fetus.^[4]^

Second, the length of placental support and a prolonged duration of airway management using devices, including the GVL, during the EXIT procedure cannot be ignored. Prolonged duration of the EXIT procedure along with uterine relaxation with a high concentration (often exceeding 2 MAC) of volatile anesthetic agents and continuous intravenous infusion of nitroglycerin for maintaining uteroplacental circulation can result in prolonged uterine atony and continued postpartum hemorrhage.^[12]^ In such situations, it is necessary to prepare sufficient blood products for large amounts of blood loss, and oxytocin in conjugation with uterine massage may be needed to recover uterine contraction.^[12]^ Fortunately, the EXIT procedure with uteroplacental circulation for up to 3 hours has been performed without significant maternal or fetal complications,^[19]^ and a slight increase in the time of intubation because of the use of a VL would not cause failed intubation or endanger the fetus.

Finally, other modalities for fetal intubation, including rigid bronchoscopy and even surgical tracheostomy, should be prepared in case of failed intubation with a VL. As a multidisciplinary team approach, different specialists should be on standby within the surgical field during the procedure. Neonatologists should be on standby for resuscitation, and otolaryngologist should be on standby for rigid bronchoscopy, surgical tracheostomy, or even removal of the neck mass. Depending on the fetal neck lesion, there might be difficulty in tracheal intubation and inability to achieve a laryngeal view with a VL, even for experienced physicians. A recent study found that the strongest predictor of GVL failure was altered neck anatomy resulting from a surgical scar, radiation changes, or a mass, and the authors suggested that alternative techniques of tracheal intubation should be maintained with competency.^[20]^ In our case, the GVL was primarily used, as it was believed to be the most appropriate option among all the noninvasive modalities for tracheal intubation in such fetuses. If this approach failed, rigid bronchoscopy was planned subsequently as a noninvasive option. Additionally, if all the noninvasive approaches failed, surgical tracheostomy by an otolaryngologist was planned. Although fetal intubation was successful in our case with a neck mass, there is a possibility of intubation failure using the GVL during the EXIT procedure in a fetus, as the presence of a neck mass is considered as the strongest predictor of GVL failure.

In conclusion, the GVL approach may be a useful noninvasive approach for establishing a clear fetal airway successfully during the EXIT procedure in a fetus with a large neck mass, in whom airway difficulty and subsequent airway crisis related to airway obstruction are predicted at delivery.
